# Characterisation of Elevenin-Vc1 from the Venom of *Conus victoriae*: A Structural Analogue of α-Conotoxins

**DOI:** 10.3390/md21020081

**Published:** 2023-01-25

**Authors:** Bankala Krishnarjuna, Punnepalli Sunanda, Jeffrey Seow, Han-Shen Tae, Samuel D. Robinson, Alessia Belgi, Andrea J. Robinson, Helena Safavi-Hemami, David J. Adams, Raymond S. Norton

**Affiliations:** 1 Medicinal Chemistry, Monash Institute of Pharmaceutical Sciences, Monash University, Parkville, VIC 3052, Australia; 2 Illawarra Health and Medical Research Institute (IHMRI), University of Wollongong, Wollongong, NSW 2522, Australia; 3 School of Chemistry, Monash University, Clayton, VIC 3800, Australia; 4 Department of Biochemistry, University of Utah, Salt Lake City, UT 84112, USA

**Keywords:** NMR, three-dimensional structure, nicotinic acetylcholine receptors

## Abstract

Elevenins are peptides found in a range of organisms, including arthropods, annelids, nematodes, and molluscs. They consist of 17 to 19 amino acid residues with a single conserved disulfide bond. The subject of this study, elevenin-Vc1, was first identified in the venom of the cone snail *Conus victoriae* (*Gen. Comp. Endocrinol*. **2017**, *244*, 11–18). Although numerous elevenin sequences have been reported, their physiological function is unclear, and no structural information is available. Upon intracranial injection in mice, elevenin-Vc1 induced hyperactivity at doses of 5 or 10 nmol. The structure of elevenin-Vc1, determined using nuclear magnetic resonance spectroscopy, consists of a short helix and a bend region stabilised by the single disulfide bond. The elevenin-Vc1 structural fold is similar to that of α-conotoxins such as α-RgIA and α-ImI, which are also found in the venoms of cone snails and are antagonists at specific subtypes of nicotinic acetylcholine receptors (nAChRs). In an attempt to mimic the functional motif, Asp-Pro-Arg, of α-RgIA and α-ImI, we synthesised an analogue, designated elevenin-Vc1-DPR. However, neither elevenin-Vc1 nor the analogue was active at six different human nAChR subtypes (α1β1εδ, α3β2, α3β4, α4β2, α7, and α9α10) at 1 µM concentrations.

## 1. Introduction

Cone snail venoms are a rich source of toxins, including many hormone-like peptides [1,2,3]. These peptides typically function by targeting membrane proteins such as ion channels, transporters, and G-protein-coupled receptors (GPCRs) [4,5,6]. New classes of conopeptides continue to attract interest as potentially new pharmacological tools or therapeutic leads. This is exemplified by cone snail insulins, which are active at the human insulin receptor [7,8,9], and recently discovered somatostatin analogues in the venom of fish-hunting cone snails [10]. The focus of this study, elevenin-Vc1, was discovered in the venom of the mollusc-hunting cone snail *Conus victoriae* [1,3], but its structure, function, and physiological target have not yet been characterised.

The elevenins are a class of peptides identified in a wide range of organisms, including the venom of cone snails [3,11,12]. However, they are understudied with respect to both their structure and function. Elevenin was first identified in the L11 abdominal ganglion [11] and the head ganglia [13] of the sea hare, *Aplysia californica* [14]. Immunochemistry studies found the elevenin-precursor peptide in the salivary gland, suggesting that this gland could be another neurosecretory tissue region in addition to the brain [15,16]. A range of biological activities has been reported for elevenin peptides in different organisms. In the nematode *Caenorhabditis elegans* elevenin suppresses olfactory plasticity, thus regulating population density as the plasticity of olfactory behaviour is dependent on population density [17]. Elevenin from the annelid polychaete worm *Platynereis dumerilii* was found to be a neurotransmitter [18]. Bauknecht and Jekely identified *P. dumerilii* elevenin activity against two orphan receptors (elevenin receptors) [19] which belong to a group of related GPCRs that are generally activated by ligands containing one or two disulfide bonds [20]. Studies on the brown planthopper insect *Nilaparvata lugens* identified the expression of elevenin, mainly in the brain, abdominal integument, and salivary glands; knockdown of elevenin or its receptor NlA42 expression by RNA interference caused melanisation [16,21]. In the red flour beetle, *Tribolium castaneum*, knockdown of the elevenin receptor caused mortality during the larval stage [22]. Elevenin regulates cuticle melanisation in *N. lugens* through the tyrosine-mediated cuticle melanism pathway [16,21]. It is also involved in salivary secretion and the early stages of ovarian development in the black-legged tick, *Ixodes scapularis* [15]. Despite these extensive functional studies, no structural information is available on these peptides. 

Elevenin-Vc1 was identified in the venom of *C. victoriae* (Uniprot id: A0A0F7YZQ7) [3]. Based on its amino acid sequence similarity to the other elevenins, elevenin-Vc1 was predicted to mimic the function of the prey’s neuropeptide but was not characterised functionally, and there is no structural information available on this peptide or other members of the elevenin family. The structure of this peptide would be useful to understand its interaction with the receptor(s) and its physiological function. In this study, we have determined the solution structure of elevenin-Vc1 using nuclear magnetic resonance (NMR) spectroscopy and investigated its activity in mice and against different human nicotinic acetylcholine receptors (nAChRs).

## 2. Results and Discussion

### 2.1. Elevenin-like Peptides Are Widely Distributed in Protostomes

**Figure 1:**
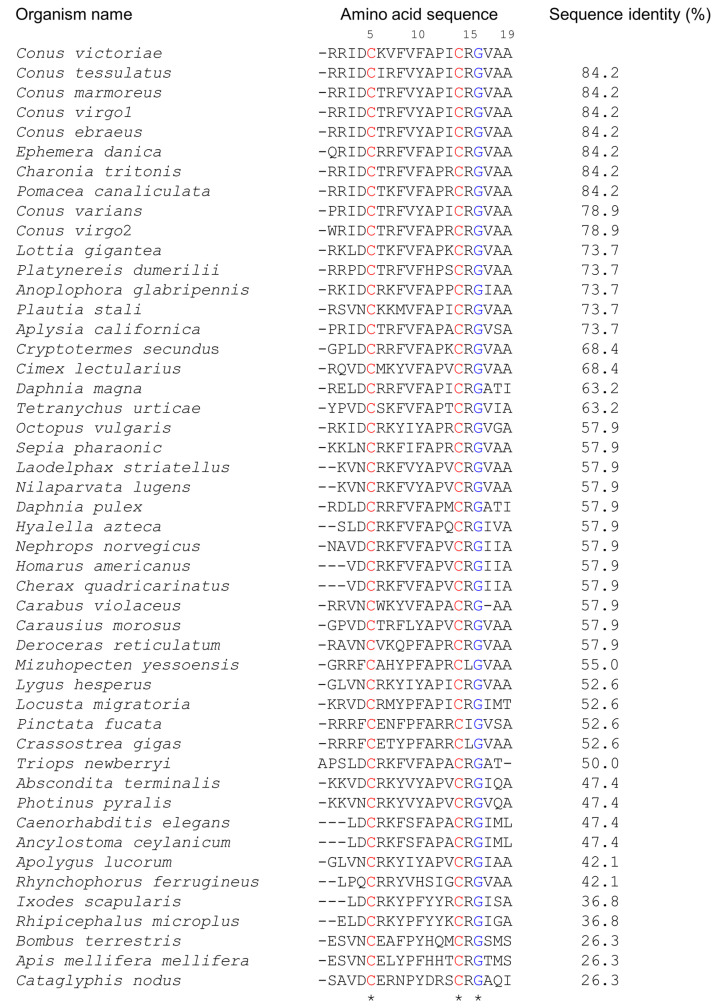
Alignment of the amino acid sequence of elevenin-Vc1 with the sequences of elevenin peptides identified from different organisms. The sequence alignment was performed in Clustal Omega and the pairwise alignment to estimate the sequence identity of elevenin-Vc1 with other sequences was performed using EMBOSS Needle. The numbering is based on the elevenin-Vc1 sequence. *C. victoriae* (Uniprot id: A0A0F7YZQ7; Uniprot IDs for other sequences are in parentheses), Conus tessulatus*, Conus marmoreus*, *Conus virgo*1*, Conus ebraeus*, *Ephemera danica* (green drake mayfly; A0A6L5CU93), *Charonia tritonis* (snail; A0A1S6JQ53), *Pomacea canaliculata* (golden apple snail; A0A2T7PKS3), Conus varians*, Conus virgo2*, *Lottia gigantea* (giant owl limpet; V3ZNG6), *Platynereis dumerilii* (annelid; F8UKT2), Anoplophora glabripennis* (Asian long-horned beetle), *Plautia stali* (stink bug; A0A1E1G7S6), *Aplysia californica* (sea hare; P06518), *Cryptotermes secundus* (drywood termite; A0A2J7Q147), *Cimex lectularius* (bed bug; A0A8I6S9P9), *Daphnia magna* (crustacean; A0A164LM77), *Tetranychus urticae* (two-spotted spider mite; T1JRJ2), *Octopus vulgaris* (common octopus; A0A6P7SM57), *Sepia pharaonic* (Pharaoh cuttlefish; A0A812CC38), *Laodelphax striatellus* (small brown planthopper; A0A482WKF4), *Nilaparvata lugens* (brown planthopper; U3U8R9), *Daphnia pulex* (water flea; E9FTB5), *Hyalella azteca* (amphipod; A0A8B7N5M0), *Nephrops norvegicus* (Norway lobster; A0A4D6BQ19), *Homarus americanus* (crustacean; A0A8J5JXL8), *Cherax quadricarinatus* (Australian red claw crayfish; A0A2U8JAF9), *Carabus violaceus* (violet ground beetle; A0A7U3RBJ9), *Carausius morosus* (Indian stick insect; A0A6G4ZWF7), *Deroceras reticulatum* (grey garden slug; A0A1X9WEC4), *Mizuhopecten yessoensis* (Japanese scallop; A0A210R6A6), *Lygus hesperus* (western plant bug; A0A7U0K8Q2), *Locusta migratoria* (migratory locust; A0A0H3YJD5), Pinctata fucata* (Akoya pearl oyster), *Crassostrea gigas* (Pacific oyster; K1QWX1), Triops newberryi* (desert tadpole shrimp), *Abscondita terminalis* (beetle; A0A7J7BRH2), *Photinus pyralis* (common eastern firefly; A0A5N4A793), *Caenorhabditis elegans* (nematode; Q11099), *Ancylostoma ceylanicum* (hookworm; A0A016W9Q8), *Apolygus lucorum* (plant bug; A0A6A4J2V7), *Rhynchophorus ferrugineus* (red palm weevil; A0A5Q0TWY8), *Ixodes scapularis* (blacklegged tick; A0A346B4H7), Rhipicephalus microplus* (Asian blue tick), *Bombus terrestris* (buff-tailed bumblebee; A0A1L1WJA9), *Apis mellifera mellifera* (German honeybee; A0A184TJC4), and *Cataglyphis nodus* (ant; A0A8A4ZRY6). Elevenin sequences from *H. americanus* and *Cherax quadricarinatus*, *Caenorhabditis elegans* and *A. ceylanicum*, and *L. striatellus* and *N. lugens*, respectively, are identical. The sequences from *Conus virgo*, *Conus ebraeus,* and *Conus tessulatus* were identified from venom gland transcriptomes. * Uniprot ids are not available. The conserved Cys and Gly residues are highlighted in red and blue, respectively.

Elevenins are widely distributed in diverse organisms such as molluscs, nematodes, annelids, and arthropods (Figure 1). They are 17–19 amino acid residues in length, with two conserved Cys residues at positions 5 and 14 and a Gly residue at position 16 (numbering is based on the elevenin-Vc1 sequence). Arg15 is the next most highly conserved residue (93%), followed by Ala11 (85%), Ala19 (83%), and Pro12 (81%). The presence of similar peptides to elevenin-Vc1 in several other cone snails and molluscs, as documented in Figure 1, suggests that this class of peptide may have a common function across these species.

### 2.2. Peptide Synthesis, Oxidative Folding, Purification, and Mass Spectroscopy

Elevenin-Vc1 was produced by solid-phase peptide synthesis (SPPS). Peptide oxidation was performed in 50 mM HEPES (pH 7.25), with a final yield of ~10%. The observed mass of the oxidised peptide (Figure S1) was two Da less than that of the non-oxidised peptide, confirming the monomeric state of the peptide with one disulfide bond formed. When the crude elevenin-Vc1 was subjected to oxidative folding in 100 mM NH_4_HCO_3_, peptide dimers formed through intermolecular disulfide bonds.

### 2.3. Sequential Assignments of NMR Apectra of Elevenin-Vc1

A pH titration on elevenin-Vc1 was performed to identify the optimal pH for NMR-based structural studies. One-dimensional proton (1D ^1^H) and two-dimensional total correlation spectroscopy (2D TOCSY) and 2D nuclear Overhauser spectroscopy (2D NOESY) spectra were recorded at different pH values ranging from 2 to 6.5 (Figure S2). pH-dependent chemical shift changes were observed for a few amide protons in the 1D ^1^H NMR spectra recorded at pH 2 to 4 (Figure S2). When the sample pH was increased from 4.5 to 6.5, only Ile3 showed a small upfield shift (−0.04 ppm), and the Cys14 and Ala19 amide peaks were partially overlapped, whereas the Arg2 amide peak was completely broadened (Figure S2). Despite these small chemical shift changes, the peaks were well-resolved at pH 4.5 and the dispersion of amide proton chemical shifts was not substantially affected by varying the pH from 4.5 to 6.5, indicating good conformational stability of the peptide in this pH range. However, slight peak broadening was observed at higher pH as a consequence of the pH-dependent peak broadening that generally occurs at a higher temperature in peptides [23]. Therefore, the NMR spectra for sequential NMR assignments and the structure calculation were acquired at pH 4.5 and 283 K (Figure S3).

**Figure 2:**
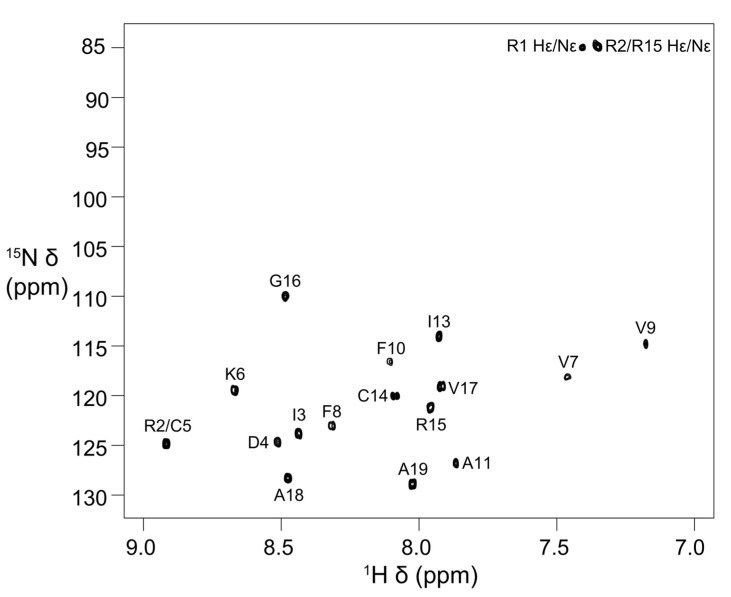
Two-dimensional [^15^N–^1^H]-HSQC spectrum of elevenin-Vc1 showing backbone amide resonance assignments. The spectrum was acquired at 303 K and pH 4 on a Bruker Avance III 600 MHz spectrometer.

Stereo-specific assignments for Hβ protons of Asp4, Cys5, Phe8, Phe10, and Cys14 and methyl groups of Ile3 were determined using 2D DQF–COSY, 2D NOESY (mixing time 50 ms) and 2D TOCSY spectra. The peaks for the Ile13 methyl groups have degenerate chemical shifts (Table S1). In addition, the Val7 and Val9 methyl proton resonances have upfield chemical shifts (Table S1 and Figure S3). The ^15^N chemical shifts were obtained from 2D [^13^C–^1^H]-HSQC and [^15^N–^1^H]-HSQC spectra, respectively. The chemical shifts have been deposited in BioMagResBank (BMRB id: 31054) [24]. The [^15^N–^1^H]-HSQC spectrum with all the backbone ^15^N–^1^H assignments and Arg side-chain Nε-Hε cross-peaks labelled with assignments is shown in Figure 2. The amide cross-peaks of Val7, Val9, and Phe10 were very weak in spectra recorded at 278 K (pH 4) and 283 K (pH 4.5), but became prominent at 303 K, suggesting the presence of a local conformational exchange process at lower temperatures that shifted to intermediate or fast exchange at higher temperatures (Figure 2 and Figure S4). 

### 2.4. Solution Structure of Elevenin-Vc1

**Figure 3:**
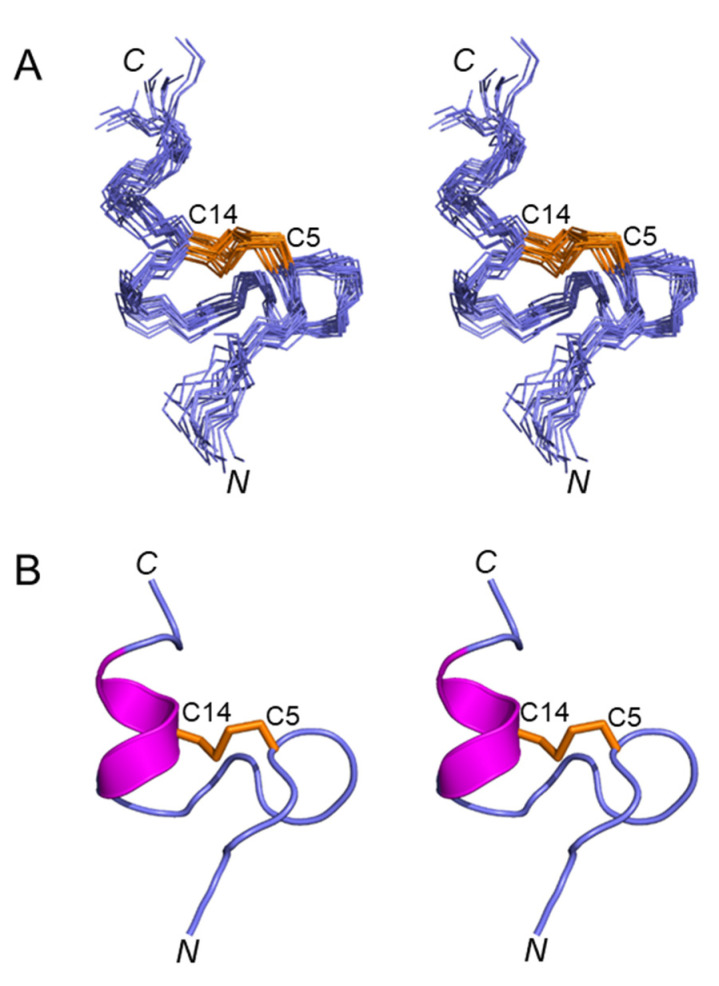
(**A**) Stereoview (cross-eyed) of the best 20 structures of elevenin-Vc1 (PDB id: 8F04) superimposed over backbone N, Cα, and carbonyl carbon for amino acid residues 1–19, with a disulfide bond. (**B**) Stereoview (cross-eyed) of the backbone of the closest-to-average structure of elevenin-Vc1. The helical region (12–15) is displayed in magenta, and the disulfide bond (Cys5–Cys14) is displayed in orange.

**Figure 4:**
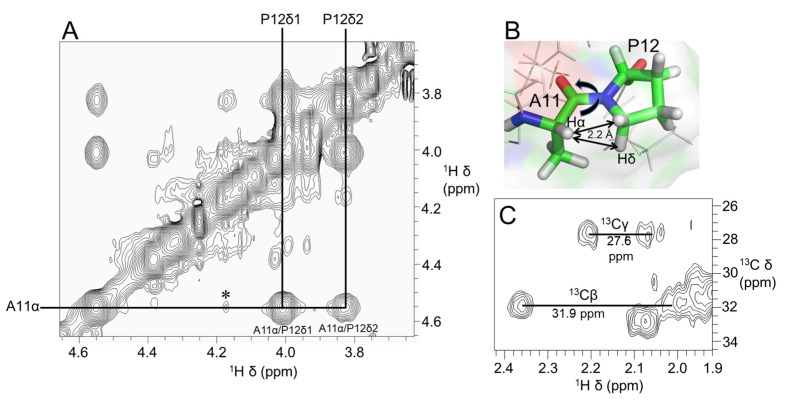
(**A**) Region of 600 MHz NOESY spectrum of elevenin-Vc1 showing NOEs diagnostic of the Ala11-Pro12 peptide bond conformation. Strong NOEs were observed between Ala11 Hα (*i*) and Pro12 Hδ protons (*i* + 1), which are ~2.2 Å apart, indicating the *trans* conformation of the Ala11-Pro12 bond (**B**). In addition, a weak NOE (⁎) is observed between Ala11 Hα and Pro12 Hα protons; this may be because these protons are within 5 Å (4.4 Å). (**C**) 2D [^13^C–^1^H]-HSQC spectrum showing Pro12 Cα and Cβ NMR assignments. The small Cβ and Cγ chemical shift difference of 4.3 ppm (<5 ppm for *trans* and ~10 ppm for *cis*) further confirms the *trans* geometry of the Ala11-Pro12 peptide bond.

Figure 3A shows a stereoview of an ensemble of 20 lowest-energy structures refined in an implicit solvent using XPLOR-NIH. Except for the N- and C-termini, the structures were well-defined, with backbone and sidechain heavy atom RMSD values of 0.4 Å and 0.8 Å, respectively, over residues 3–16 (Table 1). Elevenin-Vc1 has a five-residue bend encompassing Cys5 to Val9 and a C-terminal helix spanning residues Pro12 to Arg15 (Figure 3B). The turn and helix are connected by the disulfide bridge between Cys5 and Cys14. The N-terminal (Arg1-Ile3) and C-terminal (Gly16-Ala19) regions, and the Val9-Ala11 region between the turn and the helix, are less well defined. NOEs between the Ile13 amide proton and the Pro12 δ protons were observed, indicating that the proline is acting as a helix inducer rather than a helix breaker [25,26]. The inter-residue NOEs between the Ala11 Hα and Pro12 Hδ resonances are diagnostic of a *trans* conformation of the peptide bond between Ala11 and Pro12 (Figure 4A,B). The *trans* conformation was confirmed using Pro12 ^13^Cβ and ^13^Cγ chemical shifts (Figure 4C); the chemical shift difference between the ^13^Cβ and ^13^Cγ resonances was 4.3 ppm [27], confirming the *trans* geometry of the Ala11-Pro12 peptide bond (reference values are < 5 ppm for the *trans* conformation and ~10 ppm for *cis* [27]). The structural ensemble has been deposited in the Protein Data Bank [28] (PDB 1d: 8F04).

### 2.5. Valine-Phenylalanine Interactions

**Figure 5:**
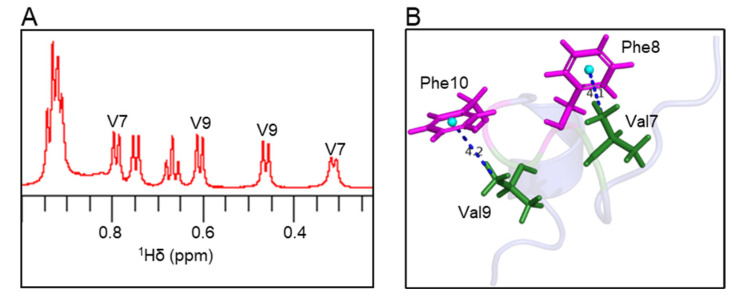
Ring current effects in the elevenin-Vc1 structure. (**A**) The expanded region of the ^1^H NMR spectrum shows methyl peaks from Val7 and Val9 residues. The Val methyl groups with unusual chemical shifts are labelled. (**B**) The methyl groups of Val7 and Val9 are oriented perpendicular to the aromatic rings of Phe8 and Phe10, respectively, and thus experience aromatic ring currents. For chemical shifts, see Table S1.

Atoms oriented perpendicular to the plane of aromatic rings in peptide/protein structures experience a shielding effect; therefore, the resonances from these atoms appear at a higher field compared to random coil chemical shifts [29,30,31]. In the ^1^H NMR spectrum, the Val7 methyl peak was shifted by −0.63 ppm, and the Val9 methyl peak was shifted by −0.48 ppm compared to the random coil chemical shifts (Figure 5A and Table S1). In the elevenin-Vc1 structure, one of the two methyl groups from Val7 and Val9 residues is oriented perpendicular to the aromatic rings of Phe8 and Phe10 residues, respectively (Figure 5B). The measured distances between the centroid of the Phe8/Phe10 aromatic ring and the Val7/Val9 methyl groups were 4.1 Å and 4.2 Å, respectively (Figure 5B).

### 2.6. Elevenin-Vc1 Made Mice Hyperactive upon Intracranial Injection

Elevenin-Vc1 was identified in the venom of *C. victoriae* [3], but its function in the venom is not known. Many conotoxins display activity in mice when injected intracranially [32]. Here we tested the effect of elevenin-Vc1 in mice by intracranial injection (Table 2). Intracranial injection of elevenin-Vc1 in mice at doses of 5 or 10 nmol caused increased activity over the 1 h period of observation compared to negative control animals, suggesting the presence of a mammalian target receptor for this peptide. The peptide did not produce any observable changes to normal or depolarisation-induced intracellular Ca^2+^ levels in cultured mouse dorsal root ganglion cells, indicating that the target receptor is not expressed in these sensory neurons (data not shown).

### 2.7. Elevenin-Vc1 Has a Structural Fold Similar to That of α-Conotoxins

**Figure 6:**
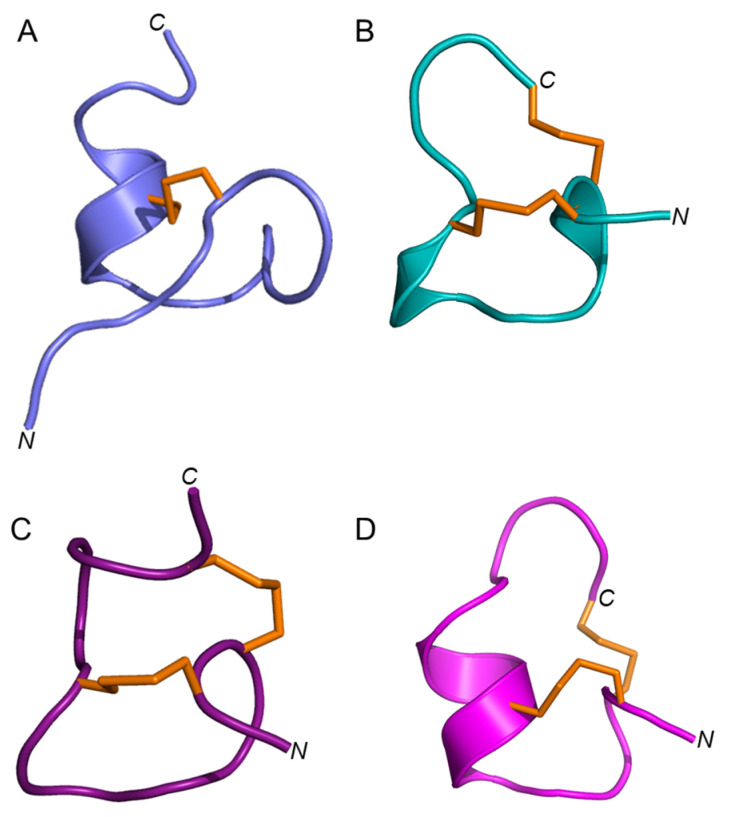
Structures of elevenin-Vc11 and α-conotoxins with the elevenin-Vc1-like structural fold. (**A**) Elevenin-Vc1 (PDB id: 8F04) (**B**) α-ImI (PDB id: 1IMI [cyan]), (**C**) α-RgIA (PDB id: 2JUT [dark red]), and (**D**) α-Vc1.1 (PDB id: 2H8S [magenta]). The disulfide bonds are displayed in orange.

**Figure 7:**
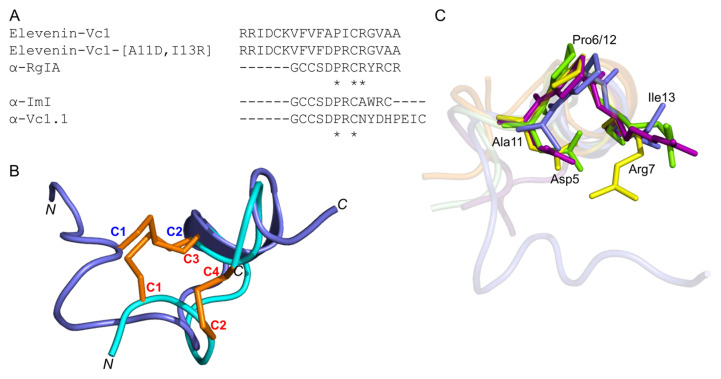
(**A**) Amino acid sequence alignment of elevenin-Vc1, α-RgIA, α-ImI, α-Vc1.1, and elevenin-Vc1-DPR. The Ala11 and Ile13 residues are mutated to Asp11 and Arg13 (highlighted in bold). * indicate the structurally conserved residues in all sequences based on a multiple sequence alignment using Clustal Omega. (**B**) Structure alignment of elevenin-Vc1 (blue) with α-ImI (PDB id: 1IMI) (cyan). The disulfide bonds (C1–C2 in elevenin-Vc1 and C1–C3, C2–C4 in α-ImI) are shown in orange. (**C**) Structure-alignment of elevenin-Vc1 (PDB id: 8F04 [blue]), α–RgIA (PDB id: 2JUT [dark-red]), α–ImI (PDB id: 1IMI [green]), and α-Vc1.1 (PDB id: 2H8S [yellow]). The amino acid residues (Asp5, Pro6, and Arg7) critical for the function of α-RgIA, α-ImI, and α-Vc1.1 are highlighted (coloured sticks) and labelled. The amino acid residues from elevenin-Vc1 (Ala11, Pro12, and Ile13) that are structurally aligned with Asp5, Pro6, and Arg7 in α-RgIA, α-ImI, and α-Vc1.1 are also highlighted with sticks and labelled. Disulfide bonds and protons are not shown for clarity.

Elevenin-Vc1 has a structural fold with some similarities to those of α-conotoxins such as α-RgIA from *C. regius* [33], α-ImI from *C. imperialis* [34], and α-Vc1.1 from *C. victoriae* [35] (Figure 6); RMSD values are shown in Table S2. Elevenin-Vc1 has a single disulfide bond (Figure 6A), whereas α-RgIA, α-ImI, and α-Vc1.1 have two (Figure 6B–D). Elevenin-Vc1 and α-RgIA sequences are conserved at three amino acid residues, whereas elevenin-Vc1, α-ImI, and α-Vc1.1 are conserved at two, including one of the Cys residues (Figure 7A). Despite their structural similarities, it is unlikely that elevenin-Vc1 and α-conotoxins evolved from a common ancestor. The gene structure of the A-superfamily conotoxins that most α-conotoxins belong to is characterised by two exons separated by a single phase 1 intron [36], whereas the elevenin gene retrieved from the genome of *Lautoconus ventricosus* is encoded by three exons separated by two phase 2 introns [37]. 

### 2.8. Elevenin-Vc1 and Elevenin-Vc1[A11D, I13R] (Elevenin-Vc1-DPR) Are not Active against nAChRs 

α-RgIA [38] and α-Vc1.1 [35] selectively block the α9α10 nAChR subtype, whereas α-ImI is an inhibitor of the α7 and α3β2 nAChR subtypes [39]. Since elevenin-Vc1 has a structural fold similar to that of α-RgIA, α-ImI, and α-Vc1.1 (Figure 7B), the peptide was tested against six different human nAChR subtypes—α1β1εδ, α3β2, α3β4, α4β2, α7, and α9α10. The activity was measured by recording the changes in ACh-evoked membrane currents in *Xenopus laevis* oocytes expressing each of these receptors (Figure S5). Elevenin-Vc1 did not inhibit any of the subtypes tested (Figure S6). In an attempt to improve the activity of elevenin-Vc1, we designed an analogue using a structure-based approach. In the structure alignment, the Asp5, Pro6, and Arg7 residues of α-RgIA, α-ImI, or α-Vc1.1 are aligned with Ala11, Pro12, and Ile13 residues in elevenin-Vc1, respectively (Figure 7C). Hence, an analogue of elevenin-Vc1, elevenin-Vc1[A11D, I13R], containing the DPR sequence (elevenin-Vc1-DPR) was generated by replacing Ala11 and Ile13 with Asp11 and Arg13 residues, respectively (Figure 7A and Figure S1), and its activity was tested against the different nAChRs mentioned above. However, elevenin-Vc1-DPR was also inactive against the human nAChRs (Figures S5 and S6). The lack of activity for elevenin-Vc1 compared to α-conotoxins may be due to the structural variations caused by sequence differences and the different loop sizes between these peptides [40,41,42]. 

The orientations of the Ala11, Pro12, and Ile13 residues in the elevenin-Vc1 structure are similar to that of Asp5, Pro6, and Arg7 residues in α-RgIA and α-ImI (Figure 7C). This similarity is intriguing given that α-RgIA and α-ImI contain an additional disulfide bond.

Most conotoxins have multiple disulfide bonds, although there are some that lack disulfide bonds [2,43,44,45,46]. Those peptides with multiple disulfide bonds are relatively stable compared to those lacking them [44]. Elevenins are slightly longer than α-conotoxins, but they share some structural similarities with that class of peptide toxins. These similarities in structure may be an example of convergent evolution, but their different targets—nAChRs for α-conotoxins and most likely GPCRs for elevenins—imply that they have evolved independently. There are some examples of α-conotoxins with single disulfide bonds. A mutant of the cyclic analogue of α-conotoxin Vc1.1 lacking one disulfide bond was shown to be similar to wild-type cyclic α-conotoxin cVc1.1 (with two disulfide bonds) in activity against human α9α10 nAChR and the Cav2.2 and Cav2.3 calcium channels [47]. The removal of the first disulfide bond (C2-C8) also eliminated the formation of multiple isomers due to disulfide bond shuffling during peptide folding. Czon1107 from *C. zonatus*, which contains a single disulfide bridge, is an allosteric, non-competitive inhibitor of hα3β4 and α7 nAChRs [48,49].

An analogue of elevenin-Vc1, L11 from *A*. *californica*, is known to act on GPCRs [20], and since elevenin-Vc1 has 73% amino acid sequence identity to L11, it will be interesting to test elevenin-Vc1 on these receptors. Information on the structure of peptide toxins alone and in complex with their receptors is valuable in guiding the development of target-specific analogues that could be useful as pharmacological tools [50,51,52,53,54]. 

## 3. Conclusions

We have described the first structure of an elevenin peptide (elevenin-Vc1), determined in solution using NMR spectroscopy. The structure has a short helix near the C-terminus stabilised by a single disulfide bond, and the structural fold is similar to the α-conotoxins such as α-RgIA, α-ImI, and α-Vc1.1. The behaviour induced by the elevenin-Vc1 in mice suggests that the peptide may act on receptors in the brain. Both elevenin-Vc1 and the analogue elevenin-Vc1-DPR lack inhibitory activity against human nAChR subtypes, but further studies focusing on the effect of elevenin-Vc1 on a range of GPCRs and other membrane protein receptors in different cell lines may provide insights into its primary target(s).

## 4. Materials and Methods

### 4.1. Peptide Synthesis

All peptides were synthesised by standard Fmoc SPPS on an automated peptide synthesizer (PS3, PTI Instruments). 0.1 mmol 2-CTC resin (0.8 mmol/g loading) was swollen in dichloromethane (DCM), and the first amino acid was coupled onto the resin for 1 h using 3-fold excess amino acid and 6-fold excess) N,N-diisopropylethylamine (DIPEA) in DCM. To cap unreacted functionalities on the resin, 2 mL methanol was added and allowed to mix for 15 min. After washing the resin with DCM and dimethylformamide (DMF), Fmoc deprotection was carried out with 20% piperidine in DMF twice for 3 min. Subsequent couplings were performed for 50 min under activation of 0.3 mmol (3 equiv) O-(1H-6-chlorobenzotriazole-1-yl)-1,1,3,3-tetramethyluronium hexafluorophosphate and 0.6 mmol (6 equiv) DIPEA.

Cleavage of completed peptides was performed in trifluoroacetic acid (TFA):triisopropylsilane (TIPS):water:phenol: 3,6-dioxa-1,8-octanedithiol (DODT) [90:2.5:2.5:2.5:2.5 (vol/vol)] for 3 h. Cleaved material was precipitated and washed with cold diethyl ether twice and collected by centrifugation at 4500 rpm for 15 min at 0 °C. The crude peptide was dissolved in 50% acetonitrile (ACN), and 0.1% TFA, and freeze-dried for storage until in vitro oxidative folding and purification.

Crude peptide at 0.3 mg/mL concentration was subjected to air oxidation in 50 mM HEPES buffer (pH 7.3) containing 50 mM NaCl for 18 h. Disulfide bond formation was confirmed by LC-MS. The reaction was centrifuged to separate non-dissolved material, or precipitate, and the supernatant was filtered and purified by reversed-phase high-performance liquid chromatography (RP-HPLC). A 5–60% linear gradient of solvent B (99.9% ACN, 0.1% TFA) against solvent A (0.1% TFA in water) over 60 min through a C18 column (Vydac, 10 × 300 mm) was used to purify the peptides. Liquid chromatography-mass spectroscopy (LC-MS) was used to assess the purity and mass of peptides (Figure S1).

### 4.2. NMR Sample Preparation

The sample for NMR experiments was prepared by dissolving lyophilised peptide at a concentration of 0.8 mM in 95% H_2_O/5% ^2^H_2_O and adjusting the pH to 4.5 by adding 0.1 M NaOH. The sample for the deuterium exchange experiment was prepared by dissolving the peptide at 0.5 mM concentration in 100% ^2^H_2_O and adjusting the pH to 4.5 (uncorrected for deuterium isotope effect) by adding 0.1 M ^2^HCl or 0.1 M NaO^2^H.

### 4.3. NMR Data Collection, Processing, and Analysis

One-dimensional ^1^H NMR spectra were recorded at 283 K, with different pH conditions ranging from 2 to 7. The two-dimensional (2D) total correlation spectroscopy (TOCSY, 80 ms and 100 ms spin lock times) and nuclear Overhauser spectroscopy (NOESY, 50 ms and 300 ms mixing times) NMR spectra were recorded at different temperatures for residue-specific and sequential NMR assignments, respectively. Double-quantum filtered correlation spectroscopy (DQF-COSY), TOCSY (30 ms spin lock time), and NOESY (50 ms mixing time) NMR spectra were recorded for stereospecific assignments of Hβ protons. [^13^C–^1^H]-HSQC and [^15^N–^1^H]-HSQC spectra were recorded for obtaining ^13^C and ^15^N chemical shifts, respectively. Spectra were processed using TopSpin (version 3.5), and chemical shifts and NOE assignments were made in CcpNmr software.

### 4.4. Structure Determination

The structure of elevenin-Vc1 was calculated using distance restraints derived from intensities of NOE cross-peaks in a 2D NOESY spectrum with a mixing time of 300 ms. In total, 246 NOE-derived distances were converted into unambiguous structural restraints. Dihedral angle restraints were estimated from ^3^*J*_HN-Hα_ coupling constants measured from 1D ^1^H or 2D DQF-COSY spectra, using the following ranges: −120 ± 40° when ^3^*J*_HN-Hα_ ≥ 8Hz and −65 ± 25° when ^3^*J*_HN-Hα_ ≤ 6Hz. Three distance restraints were used for disulfide connectivity as follows: 2, 3, and 3Å for S(i)–S(j), S(i)–Cβ(j), and S(j)–Cβ(i), respectively. No hydrogen bond restraints were included in the structure determination. Initial structures were calculated by CYANA (version 3.97) using only NOE distance constraints [55]. The structures generated using CYANA were refined by simulated annealing, first *in vacuo*, then in an implicit solvent using the EEFx force field in XPLOR-NIH (version 2.45) [56]. Ramachandran statistics were generated using MolProbity Ramachandran analysis in the protein structure validation suite version 1.5 [57]. The structure figures were generated using the PyMOL Molecular graphics system, version 2.2.0 Schrödinger, LLC (http://www.pymol.org, accessed on 4 December 2022).

### 4.5. Mice behavioural Experiments

Swiss Webster mice (14–17 days old; 6.7–8.9 g) were injected intracranially (IC) with different doses of synthetic peptide dissolved in 12 μL of 0.9% NaCl, and behaviour was observed for 1 h to determine differences between treated and control animals. The screening was performed in duplicate at an initial dose of 10 nmol and repeated at lower concentrations until there were no observable differences from negative control animals (injected with 12 μL of 0.9% NaCl). All experiments involving the use of animals were approved by the Institutional Animal Care and Use Committee of the University of Utah (IACUC #14-08018). While the peptide used in these and other functional assays was highly pure (Figure S1), concentrations were not corrected for peptide content (likely to be around 70% of the dry weight following purification by RP-HPLC).

### 4.6. Assay against Heterologous Human nAChRs Expressed in Xenopus laevis Oocytes

All procedures were approved by the University of Wollongong Animal Ethics Committee (project number AE2003). Female *Xenopus laevis* were sourced from Nasco (Fort Atkinson, WI, USA), and a maximum of four frogs were kept in a 15 L aquarium at 20–26 °C with a 12 h light/dark cycle. Oocytes were obtained from five-year-old frogs anaesthetised with 1.7 mg/mL ethyl 3-aminobenzoate methanesulfonate (pH 7.4 with NaHCO_3_). Stage V-VI oocytes (Dumont’s classification; 1200–1300 μm diameter) were defolliculated with 1.5 mg/mL collagenase Type II (Worthington Biochemical Corp., Lakewood, NJ, USA) at room temperature for 1–2 h in OR-2 solution containing (in mM): 82.5 NaCl, 2 KCl, 1 MgCl_2_ and 5 HEPES at pH 7.4. 

The human muscle nAChR clones (α1, β1, δ, and ε) were purchased from Integrated DNA Technologies (Coralville, IA, USA), the human (h) α3, α9, α10, β2, and β4 clones were purchased from OriGene (Rockville, MD, USA), and all were subsequently inserted into the pT7TS vector. The human α4 and α7 clones were obtained from Prof. Jon Lindstrom (University of Pennsylvania, Philadelphia, PA, USA). Plasmid constructs of the human nAChR clones were linearised for in vitro mRNA synthesis using mMessage mMachine transcription kit (AMBION, Forster City, CA, USA).

Oocytes were injected with 5 ng cRNA for hα1β1εδ, hα3β2, hα3β4, and hα4β2, 10 ng cRNA for hα7 nAChR, and 35 ng cRNA for hα9α10 nAChR (concentration confirmed spectrophotometrically and by gel electrophoresis). The muscle subunit cRNA ratio was 2:1:1:1 (α1:β1:ε:δ), whereas the heteromeric α and β subunit cRNA ratio was 1:1, injected using glass pipettes pulled from glass capillaries (3-000-203 GX, Drummond Scientific Co., Broomall, PA, USA). Oocytes were incubated at 18 °C in sterile ND96 solution composed of (in mM): 96 NaCl, 2 KCl, 1 CaCl_2_, 1 MgCl_2_, and 5 HEPES at pH 7.4, supplemented with 5% fetal bovine serum, 50 mg/L gentamicin (GIBCO, Grand Island, NY, USA) and 10000 U/mL penicillin-streptomycin (GIBCO).

Electrophysiological recordings were carried out 2–5 days after cRNA microinjection. Two-electrode voltage clamp recordings of *X. laevis* oocytes expressing human nAChRs were performed at room temperature (21–24 °C) using a GeneClamp 500B amplifier and pClamp9 software interface (Molecular Devices, Sunnyvale, CA, USA) at a holding potential −80 mV. Voltage-recording and current-injecting electrodes were pulled from GC150T-7.5 borosilicate glass (Harvard Apparatus, Holliston, MA) and filled with 3 M KCl, giving resistances of 0.3–1 MΩ. Due to the Ca^2+^ permeability of α9α10 nAChRs, 100 µM BAPTA-AM incubation was carried out before recording to prevent the activation of *X. laevis* oocyte endogenous Ca^2+^-activated chloride channels. Oocytes expressing hα9α10 nAChRs were perfused with ND115 solution containing (in mM): 115 NaCl, 2.5 KCl, 1.8 CaCl_2_, and 10 HEPES at pH 7.4, whereas oocytes expressing all other nAChR subtypes were perfused with ND96 solution using a continuous Legato 270 push/pull syringe pump perfusion system (KD Scientific, Holliston, MA, USA) at a rate of 2 mL/min in an OPC-1 perfusion chamber of < 20 µL volume (Automate Scientific, Berkeley, CA, USA). 

Initially, oocytes were washed briefly with ND115/ND96 solution, followed by three applications of acetylcholine (ACh) at a half-maximal excitatory ACh concentration (EC_50_) for the nAChR subtypes (3 µM for hα4β2, 5 µM for hα1β1δε, 6 µM for hα3β2 and hα9α10, 100 µM for hα7 and 300 µM for hα3β4) [58]. Washout with bath solution was done for 3 min between ACh applications. Oocytes were incubated with peptides for 5 min with the perfusion system turned off, followed by co-application of ACh and compound with flowing bath solution. All peptide solutions were prepared in ND115/ND96 + 0.1% bovine serum albumin (BSA). Incubation with 0.1% BSA was performed to ensure that the BSA and the pressure of the perfusion system had no effect on nAChRs. Peak current amplitudes before (ACh alone) and after (ACh + peptide) compound incubation were measured using Clampfit version 10.7.0.3 software (Molecular Devices, Sunnyvale, CA, USA), where the ratio of ACh + peptide-evoked current amplitude to ACh alone-evoked current amplitude was used to assess the activity of the compounds at the nAChRs. All electrophysiological data were pooled (*n* = 6–8) and represent means ± standard deviation (SD). Data analysis was performed using GraphPad Prism 5 (GraphPad Software, La Jolla, CA, USA).

## Figures and Tables

**Table 1:** NMR and refinement statistics for elevenin-Vc1.

NMR Distance and Dihedral Constraints Used in XPLOR-NIH	
Total NOEs	246
Intra-residue	68
Inter-residue	184
Sequential	98
Medium-range	74
Long-range	12
Total dihedral angles	9
Backbone (ϕ angle)	7
Structure Statistics	
NOE RMSD (Å)	0.05
Angle (°)	0.5
Bonds (Å)	0.006
Improper (°)	0.18
Number of NOE violations (Å)	0
Number of angle violations	0
RMSD between 20 conformers	
Average pairwise RMSD for residues 3–16 (Å)	
Backbone (Å) (N, Cα, C)	0.40
All heavy atoms (Å)	0.80
*Ramachandran analysis*	
Amino acid residues in most favoured regions (%)	99.4
Amino acid residues in additionally allowed regions (%)	0.6

**Table 2:** Summary of results from intracranial mouse injections. Mice were 14–17 days old and weighed 6.7–8.9 g. Injections were carried out in duplicate unless stated otherwise.

Peptide	Dose [nmol]	Observed Behaviour (Time = Approximate Min Post-Injection)
Saline Control	0 (n = 4)	Normal: moving around immediately after injection, exploring the cage with intermittent grooming and resting behaviour, no jumping within first 45 min post-injection
Elevenin-Vc1	10	0–1 min: stiff tail, 0–5 min: resting position, 5–60 min: hyperactive, intensive grooming and jumping with no resting
	5	0–5 min normal behaviour, 5–60 min: hyperactive, intensive grooming and jumping with little resting
	2.5	No difference from the control mice

## Data Availability

Elevenin-Vc1 NMR chemical shifts and their structure are deposited in BioMagResDataBank (id number: 31054) and Protein Data Bank (id number: 8F04), respectively.

## Supplementary Materials

The following supporting information can be downloaded at: https://www.mdpi.com/article/10.3390/md21020081/s1, Table S1: Chemical shifts for ^1^H and ^15^N resonances of elevenin-Vc1; Table S2. Calculated RMSD values between elevenin-Vc1 and α-conotoxins; Figure S1: LC-MS profiles of elevenin-Vc1 and elevenin-Vc1-DPR; Figure S2: One-dimensional ^1^H NMR spectra of elevenin-Vc1 recorded at different pH (2 to 6.5); Figure S3: 1D ^1^H and 2D DQF-COSY, TOCSY and NOESY NMR spectra of elevenin-Vc1; Figure S4: 2D [^15^N–^1^H]-HSQC NMR spectra of elevenin-Vc1 at different temperatures; Figure S5: Representative superimposed ACh-evoked currents mediated by human AChR subtypes; Figure S6: The effect of elevenin-Vc1 and elevenin-Vc1-DPR on six human nAChR subtypes.
