# Supplementary material for: Characterisation of Elevenin-Vc1 from the Venom of Conus victoriae: A Structural Analogue of α-Conotoxins
Source: Mar Drugs. 2023 Jan 25;21(2):81. doi: 10.3390/md21020081 (PMC9963005; doi:10.3390/md21020081)
Supplement: Supplementary file 1 [file marinedrugs-21-00081-s001.zip › marinedrugs-2108573-supplementary.pdf]

Supplementary Information

Characterisation of Elevenin-Vc1 from the Venom of *Conus victoriae*: A Structural Analogue of α-Conotoxins

Bankala Krishnarjuna,[1] Punnepalli Sunanda,[1] Jeffrey Seow,[1] Han-Shen Tae,[2] Samuel D. Robinson,[1] Alessia Belgi,[3] Andrea J. Robinson,[3] Helena Safavi-Hemami,[4] David J Adams,[2] and Raymond S. Norton[1*]

[1]*Medicinal Chemistry, Monash Institute of Pharmaceutical Sciences, Monash University, Parkville 3052, Australia*
[2]*Illawarra Health and Medical Research Institute (IHMRI), University of Wollongong, Wollongong, NSW 2522, Australia*
[3] *School of Chemistry, Monash University, Clayton 3800, Victoria, Australia*
[4]*Department of Biology, University of Utah, Salt Lake City, UT 84112, USA*

*Correspondence to: Professor Raymond S. Norton, Medicinal Chemistry, Monash Institute of Pharmaceutical Sciences, Monash University, 381 Royal Parade, Parkville, Victoria 3052, Australia. Phone: (+61 3) 9903 9167. E-mail: ray.norton@monash.edu

Table S1. Chemical shifts (ppm) for ^{1}H and ^{15}N resonances of elevenin-Vc1. Spectra collected on 1 mM elevenin-Vc1 in H_2O at pH 4.5 and 283 K. n.a., not assigned. The ^{1}H chemical shifts were calibrated using dioxane peak at 3.75 ppm whereas the ^{15}N chemical were referenced indirectly. The chemical shifts were deposited into BioMagResBank with id: 31054.

	H^N	N^H	Hα	Hβ	Others
R1	n.a.[a]	n.a.[a]	4.03	1.72, 1.90	1.64,1.61 3.21,3.21 7.25
R2	8.87	124.7	4.39	1.79, 1.79	1.64,1.64 3.20,3.20 7.28
I3	8.41	124.4	4.04	1.72	1.36,1.06,0.75,0.67
D4	8.54	124.5	4.70	2.62, 3.07	
C5	8.90	125.3	4.69	2.84, 3.22	
K6	8.58	119.1	4.16	1.82, 1.95	1.50,1.39 1.68,1.68 2.99,2.99
V7	7.25		3.83	1.76	**0.31**,0.79
F8	8.23	123.0	4.72	2.71, 3.18	7.37, 7.42
V9	6.95	113.6	3.75	1.83	0.61,**0.46**
F10	8.26	116.4	4.67	2.97, 3.37	7.26, 7.36
A11	7.75	126.8	4.55	1.49	
P12			4.17	2.02, 2.36	2.21,2.07 3.83,4.01
I13	8.01	114.5	4.25	1.94	0.92,1.52,1.33 0.92
C14	7.96	120.0	4.75	2.99, 3.19,	
R15	7.80	121.3	4.34	1.85, 1.96	1.70,1.70 3.21,3.21 7.24
G16	8.44	110.0	3.93, 4.01		
V17	7.83	119.1	4.13	2.08	0.92,0.94
A18	8.46	128.6	4.32	1.38	
A19	7.99	129.2	4.09	1.32	

[a] Peak broadening due to rapid exchange with H_2O protons.
Upfield chemical shifts of Val7 and Val9 methyl groups are highlighted in bold.

Table S2. Calculated RMSD values between elevenin-Vc1 and α-conotoxins.

PDB id (peptide residues)	N+Cα+CO RMSD (Å)
Elevenin-Vc1 (8-18)/ α-ImI 1IMI (1-11)	0.233
Elevenin-Vc1 (8-15)/ α-RgIA 2JUT (1-9)	0.212
Elevenin-Vc1 (9-19/ α-Vc1.1 2H8S (1-13)	0.157

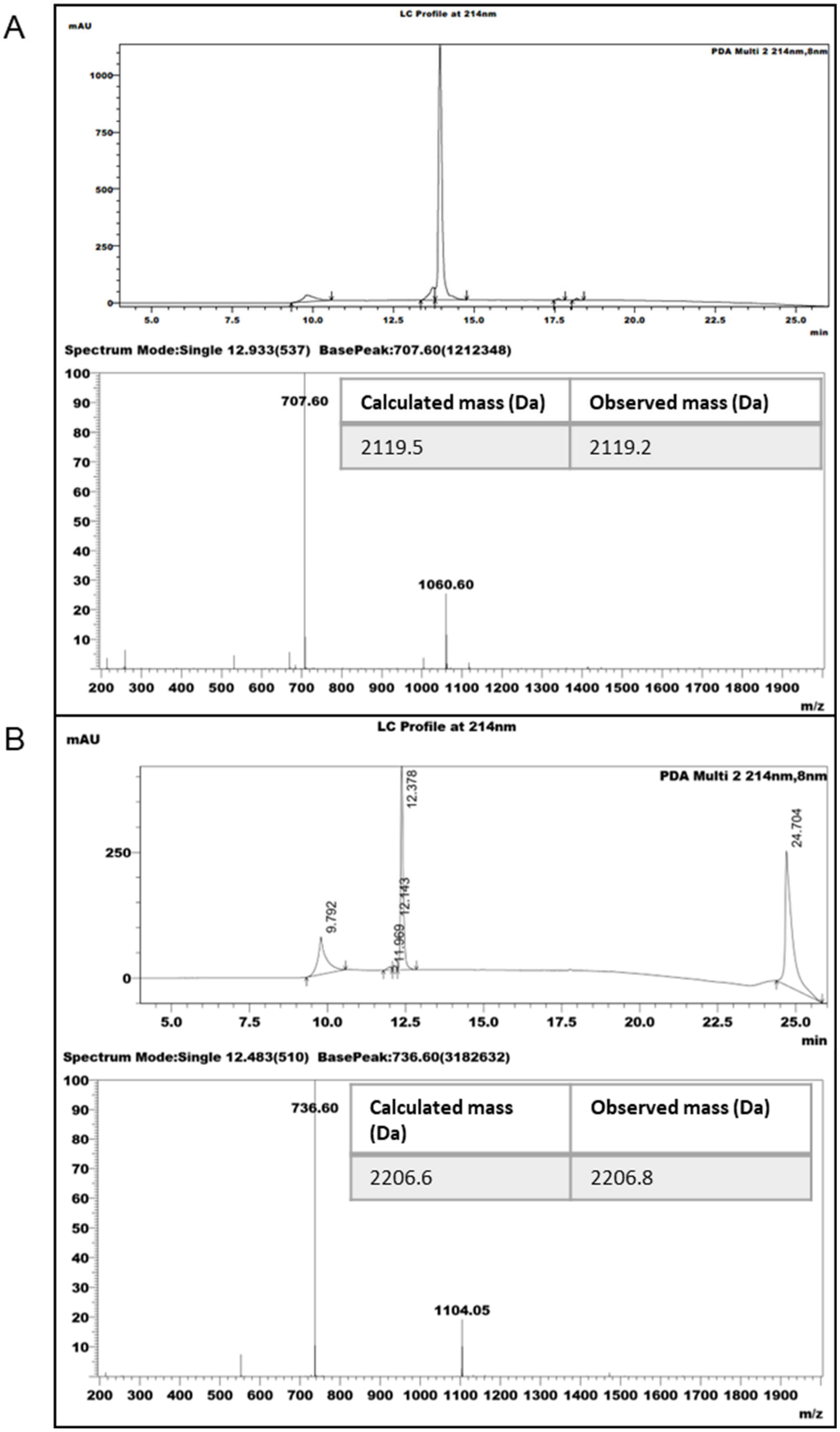

Figure S1. LC-MS profiles of (**A**) elevenin-Vc1 and (**B**) elevenin-Vc1-DPR. The estimated purity of HPLC purified peptides was ~99 %.

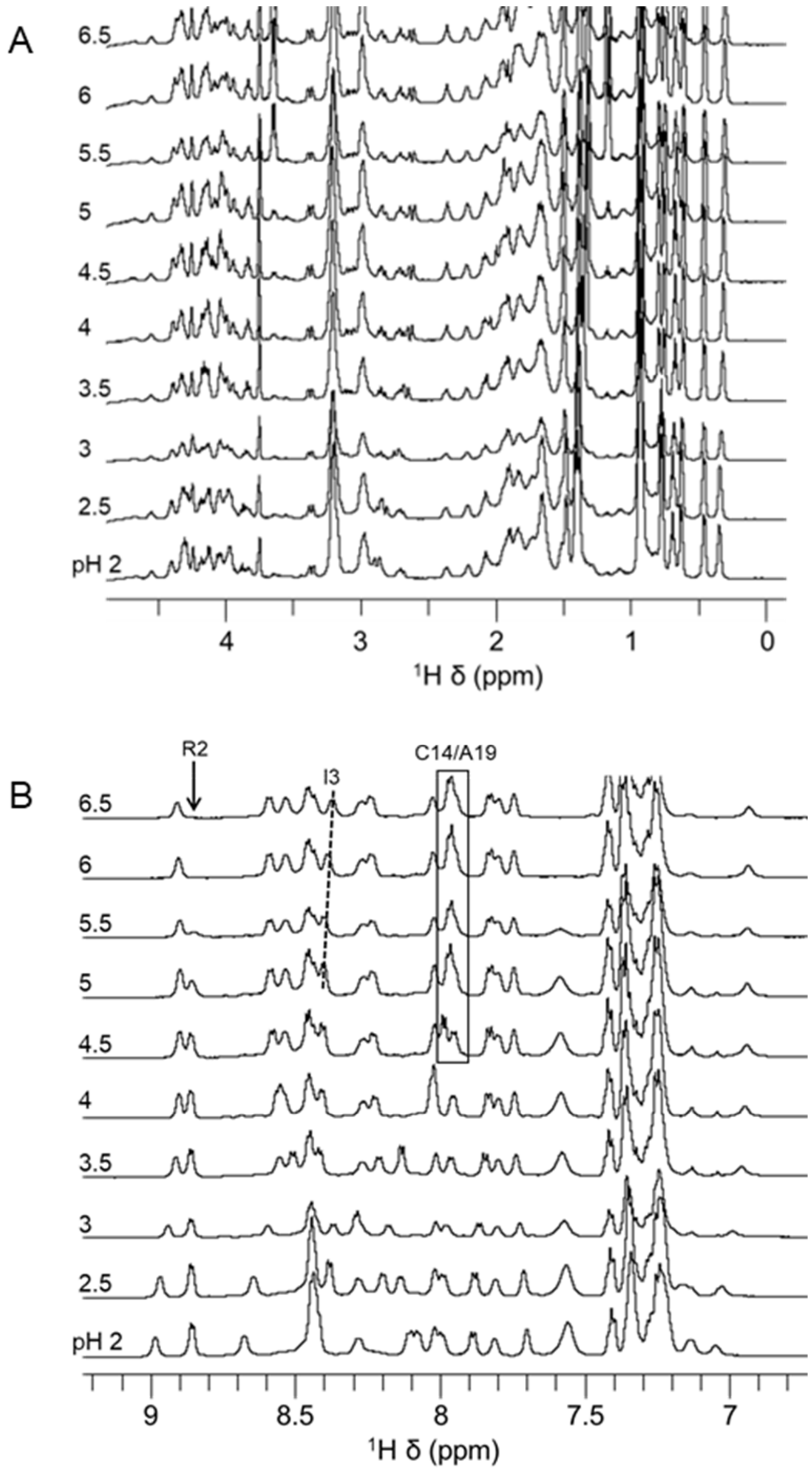

Figure S2. One-dimensional ^{1}H NMR spectra of elevenin-Vc1 recorded at different pH (2 to 6.5. (**A**) and (**B**) are the aliphatic and amide regions. Above pH 4.5, there are no substantial chemical shift changes observed. The chemical shift dispersion is maintained from pH 4.5 to pH 6.5, indicating good conformational stability of the peptide at these pH conditions. However, a slight peak broadening is observed at higher pH owing to pH-dependent amide proton exchange.

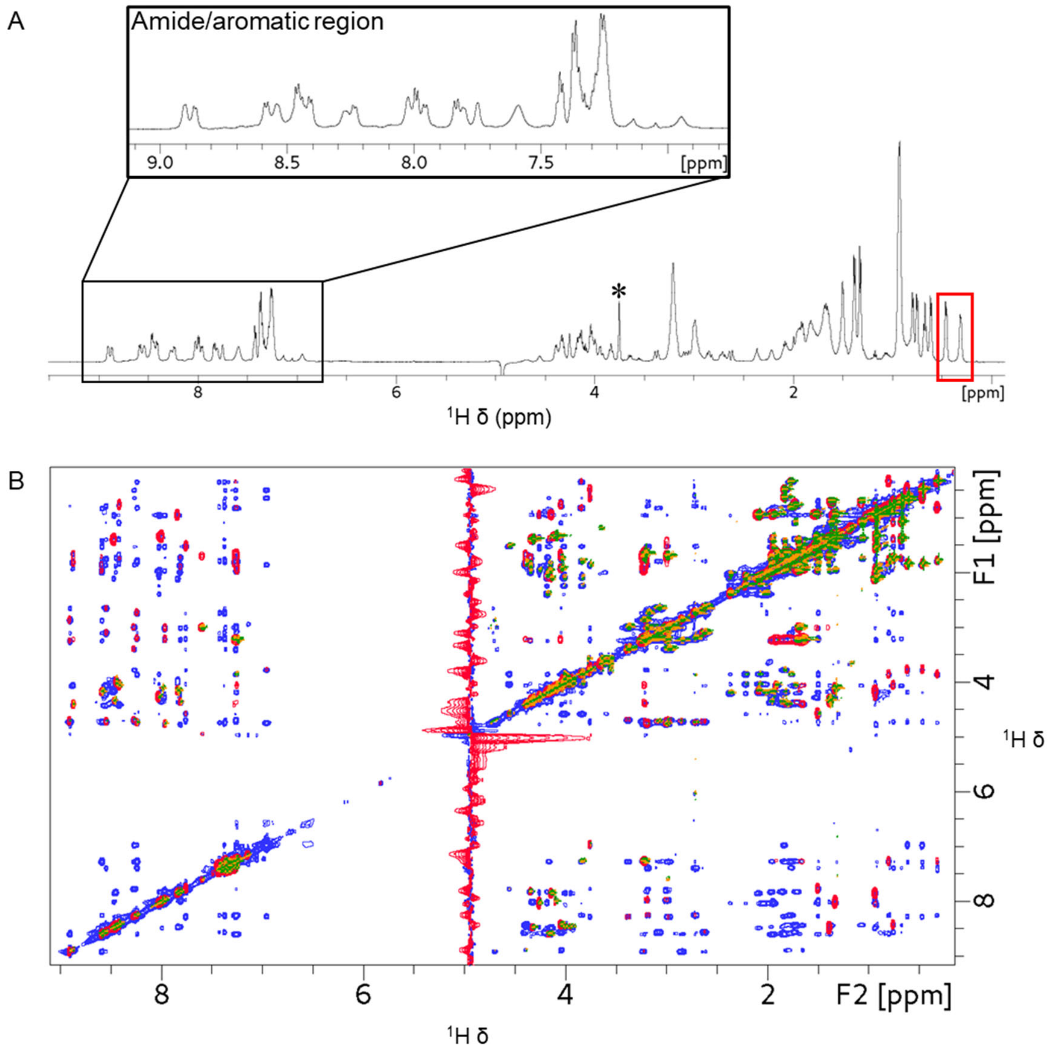

A
Amide/aromatic region
9.0
8.5
8.0
7.5
[ppm]
*
8
6
4
2
[ppm]
^{1}H δ (ppm)
B
F1 [ppm]
4
^{1}H δ
6
8
8
6
4
2
F2 [ppm]
^{1}H δ

C

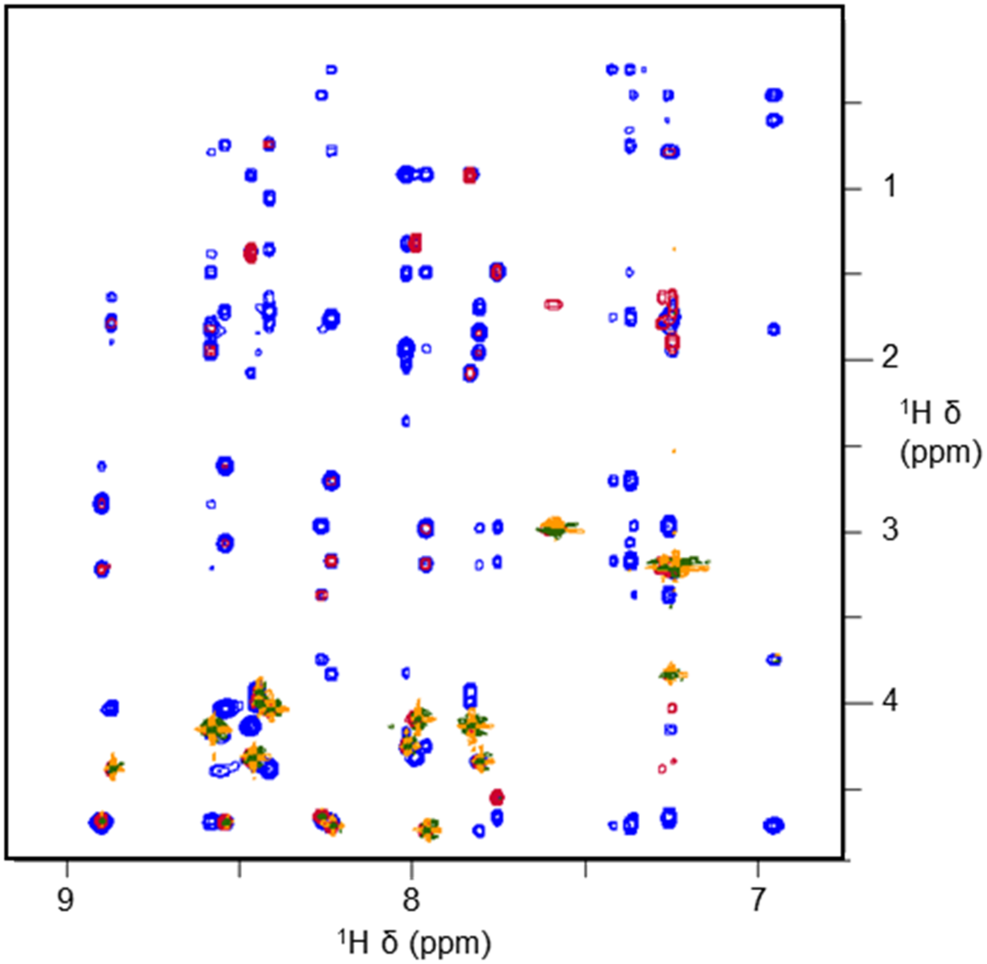

Figure S3. (**A**) One-dimensional ^{1}H-NMR spectrum of elevenin-Vc1. The amide/aromatic region is expanded for clarity. The Val7 and Val9 methyl groups with upfield chemical shifts in the high-field region are red-boxed. (**B**) Overlay of two-dimensional NOESY (blue), TOCSY (red), and DQF-COSY (green-yellow) spectra of elevenin-Vc1. (**C**) The fingerprint regions of 2D spectra in B. The spectra were recorded on a Bruker 600 MHz NMR spectrometer operated at 283 K using 0.8 mM elevenin-Vc1 peptide in 95% H_2O/ 5% $^{2}H_2O$, pH 4.5. * indicates the peak at 3.75 ppm from the reference compound, dioxane.

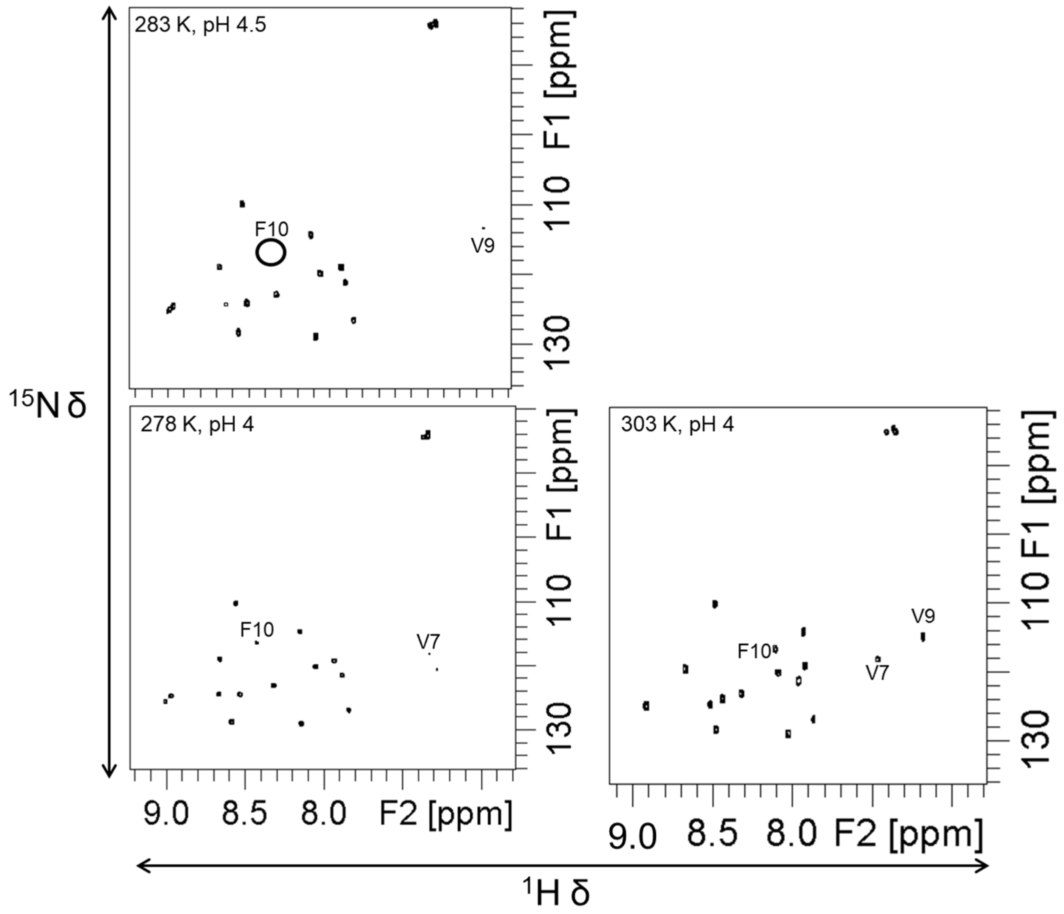

Figure S4. Two-dimensional [^{15}N-^{1}H]-HSQC spectra of elevenin-Vc1 acquired at different temperatures, as indicated. The data were collected on a Bruker Avance III 600 MHz spectrometer equipped with a cryogenically cooled probe. The peak intensities of Val7, Val9 and Phe10 amide cross-peaks are at noise level at low temperatures but become prominent at the higher temperature.

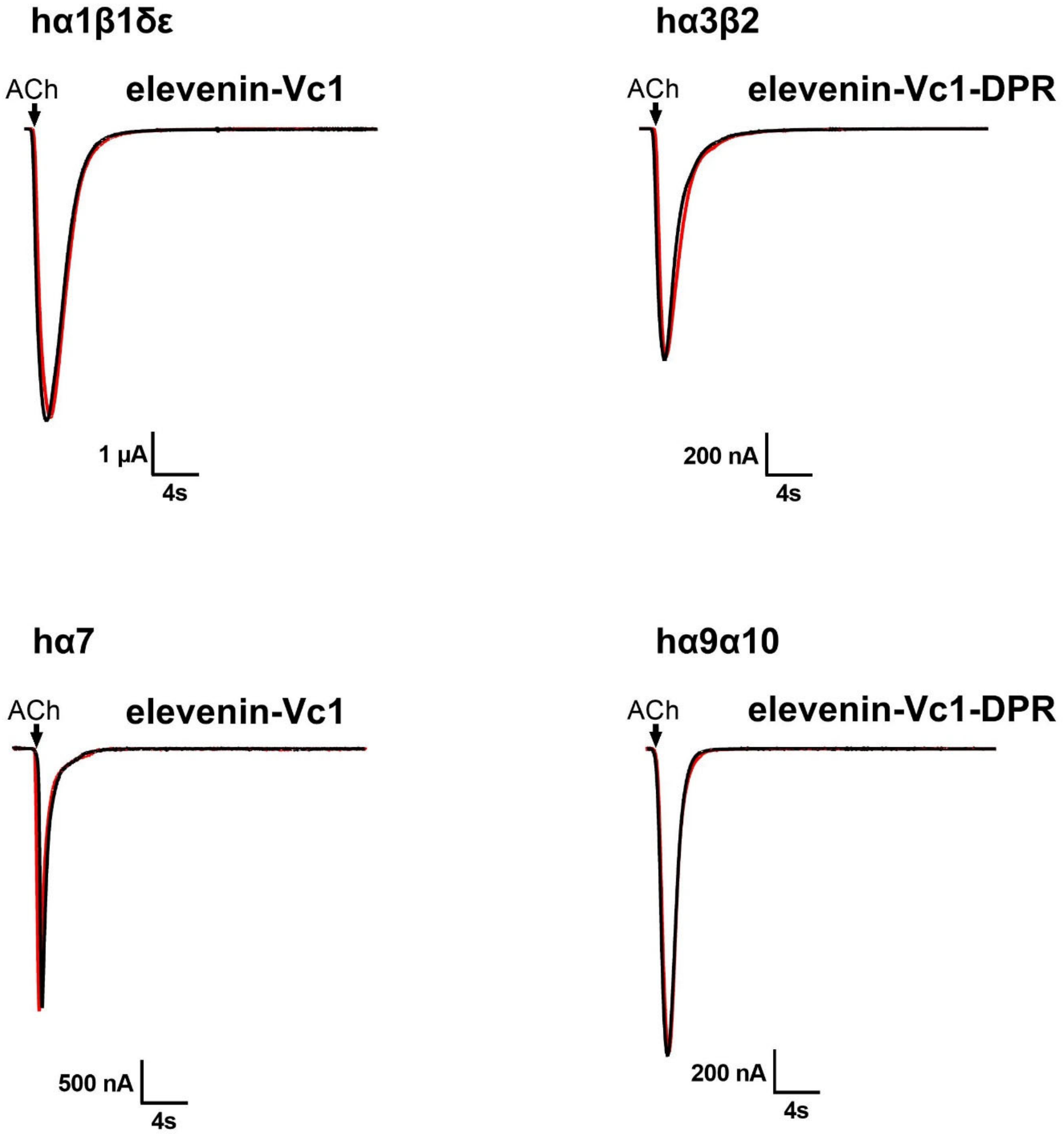

Figure S5. Representative superimposed ACh-evoked currents mediated by human (h) α1β1δε, α3β2, α7, and α9α10 nAChRs obtained in the absence (control; 5 µM, 6 µM, 100 µM and 6 µM ACh only, respectively, *black trace*) and presence of 1 µM elevenin-Vc1 or elevenin-Vc1-DPR (*red trace*).

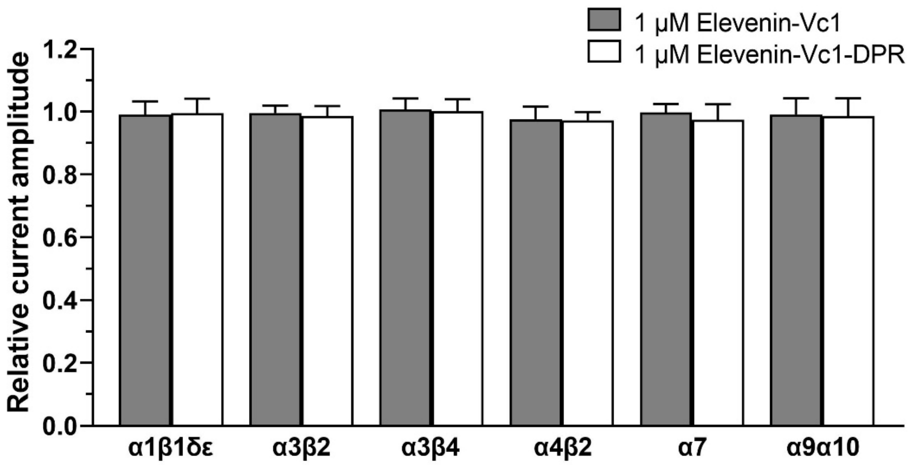

Figure S6. The lack of effect of elevenin-Vc1 and elevenin-Vc1-DPR on six different human nAChR subtypes: α1β1εδε, α3β2, α3β4, α4β2, α7 and α9α10. Bar graph of relative peak ACh-evoked current amplitude in the presence of elevenin-Vc1 or elevenin-Vc1-DPR, determined for each of the nAChR subtypes (mean ± SD, n = 6-8).
